# Altered brain state during episodic dystonia in tottering mice decouples primary motor cortex from limb kinematics

**DOI:** 10.3389/dyst.2023.10974

**Published:** 2023-02-02

**Authors:** Madelyn M. Gray, Anant Naik, Timothy J. Ebner, Russell E. Carter

**Affiliations:** 1 Department of Neuroscience, University of Minnesota, Minneapolis, MN, United States; 2 Department of Biomedical Engineering, University of Minnesota, Minneapolis, MN, United States

**Keywords:** episodic ataxia type 2, calcium imaging, generalized spike-and-wave discharges, absence seizures, P/Q-type calcium channel

## Abstract

Episodic Ataxia Type 2 (EA2) is a rare neurological disorder caused by a mutation in the *CACNA1A* gene, encoding the P/Q-type voltage-gated Ca^2+^ channel important for neurotransmitter release. Patients with this channelopathy exhibit both cerebellar and cerebral pathologies, suggesting the condition affects both regions. The *tottering (tg/tg)* mouse is the most commonly used EA2 model due to an orthologous mutation in the *cacna1a* gene. The *tg/tg* mouse has three prominent behavioral phenotypes: a dramatic episodic dystonia; absence seizures with generalized spike and wave discharges (GSWDs); and mild ataxia. We previously observed a novel brain state, transient low-frequency oscillations (LFOs) in the cerebellum and cerebral cortex under anesthesia. In this study, we examine the relationships among the dystonic attack, GSWDs, and LFOs in the cerebral cortex. Previous studies characterized LFOs in the motor cortex of anesthetized *tg/tg* mice using flavoprotein autofluorescence imaging testing the hypothesis that LFOs provide a mechanism for the paroxysmal dystonia. We sought to obtain a more direct understanding of motor cortex (M1) activity during the dystonic episodes. Using two-photon Ca^2+^ imaging to investigate neuronal activity in M1 before, during, and after the dystonic attack, we show that there is not a significant change in the activity of M1 neurons from baseline through the attack. We also conducted simultaneous, multi-electrode recordings to further understand how M1 cellular activity and local field potentials change throughout the progression of the dystonic attack. Neither putative pyramidal nor inhibitory interneuron firing rate changed during the dystonic attack. However, we did observe a near complete loss of GSWDs during the dystonic attack in M1. Finally, using spike triggered averaging to align simultaneously recorded limb kinematics to the peak Ca^2+^ response, and *vice versa*, revealed a reduction in the spike triggered average during the dystonic episodes. Both the loss of GSWDs and the reduction in the coupling suggest that, during the dystonic attack, M1 is effectively decoupled from other structures. Overall, these results indicate that the attack is not initiated or controlled in M1, but elsewhere in the motor circuitry. The findings also highlight that LFOs, GSWDs, and dystonic attacks represent three brain states in *tg/tg* mice.

## Introduction

Dystonia encompasses a spectrum of neurological disorders characterized by involuntary movements or postures resulting from sustained muscle contractions [for reviews see (1–3)]. The current classification scheme is organized along two axes (4). Axis I is based on the clinical findings and presentation. Axis II is whether the dystonia is isolated or combined, that is without or with other neurological or systemic manifestations. Just as the clinical presentations of dystonia are manifold, the etiologies are varied and range from acquired or inherited structural or degenerative nervous system lesions, drugs/toxins, and inherited forms. The prevalence of idiopathic and inherited dystonia is estimated at over 300 individuals per million (5–7).

A large number of genetic disorders have dystonia as the sole symptom or as part of the phenotype (1). Several present with episodic dystonia such as paroxysmal kinesigenic dyskinesia (PKD) due to mutations in proline-rich transmembrane protein 2 (PRRT) (8), exercise-induced dystonia owing to mutations in *SLC2A1* (*glucose transporter type 1*) (9), and dystonic episodes in *ATP1A3* spectrum disorders (10). The channelopathies are group of neurological disorders due to mutations in genes encoding for ion channels and are characterized by episodic symptoms [for reviews see (11–15)]. One of the Ca^2+^ channelopathies, and the focus of this study, results in episodic ataxia type 2 (EA2). EA2 is caused by dominant mutations of the *CACNA1A* gene that encodes the α1_A_, pore-forming subunit of the Ca_v_2.1 (P/Q-type) voltage gated Ca^2+^ channel (11, 16–20). Patients with EA2 suffer from episodic motor dysfunction consisting of limb and gait ataxia and oscillopsia, consistent with a cerebellar origin (11, 16, 17, 21). Interictally, patients may exhibit nystagmus and progressive cerebellar dysfunction linked to cerebellar atrophy (16, 17, 22). Different families have a spectrum of other episodic symptoms that suggest cerebral cortex involvement including migraine, hemiplegic paralysis, dystonia, and cognitive disturbances (11, 16, 18, 23–26). As for all channelopathies, a fundamental question is the mechanism by which a permanent abnormality in an ion channel leads to transient nervous system dysfunction (15).

The *tottering* mouse (*tg/tg*) is the most commonly used murine model for EA2 and harbors a missense mutation in *Cacna1a*, the ortholog for the human *CACNA1A* gene that results in a substitution of leucine for proline (P1802L) in the pore lining region of the P/Q-type Ca^2+^ channel (27). The *tg/tg* mouse has three major behavioral phenotypes. First, absence seizures defined by bilateral, synchronous 6–7 per second cortical generalized spike-and-wave discharges (GSWDs) in electroencephalographic recordings (EEG) accompanied by sudden movement arrest, twitching of the whiskers, and a fixed stare (28–33). Second, mild ataxia that particularly involves the hindlimbs, tail, and head movements with a wobbly gait and poorly coordinated movements (31, 34). Third, a paroxysmal motor attack, referred to as paroxysmal dyskinesia or episodic dystonia, that lasts 60–90 min with a characteristic progression along the body axis that starts in the hindlimbs (31, 35). As for the episodic cerebellar disturbances in EA2 patients, attacks in *tg/tg* mice are evoked by stress, caffeine, and ethanol (35, 36).

It is well established that the cerebellum plays a central role in the episodic dystonia by immediate early gene expression, mutant mouse models, and lesion studies (36–40). One possible mechanism underlying the attacks in *tg/tg* mice are the episodic, low-frequency oscillations (LFOs) of ~0.04–0.1 Hz, initially observed in the cerebellar cortex (41). The cerebellar LFOs occur spontaneously, can propagate to neighboring regions, last approximately 30–120 min, and are both P/Q-type channel and Ca^2+^ dependent. During the episodic dystonia, bursts of electromyographic (EMG) activity occur at the same low frequencies and are coherent with LFOs in the cerebellum (41). Similar low-frequency oscillations are also prominent in the *tg/tg* cerebral cortex (42).

We hypothesized that the LFOs provide a mechanism for generating the episodic dystonia in the *tg/tg* mouse (41, 42). While the episodic dystonia is cerebellar dependent, the presence of the LFOs in cerebral cortex as well as the role of the motor cortex in controlling movements suggests the LFOs in the cerebral cortex could contribute to the motor attacks. Importantly, two effective treatments for EA2, 4-amino pyridine (4-AP) and acetazolamide (ACTZ) (43, 44), dramatically decrease the LFOs in the cerebral cortex (42). Another striking feature of a *tg/tg* dystonic attack is the characteristic topographic progression of the simultaneous contractions of agonist and antagonist muscle groups, with the first contractions occurring in the hindlimbs, followed by spread to the torso, forelimbs, and finally the head. In the final phase, the mice regain control of their hindlimbs, while contractions of the forepaw and facial muscles continue (31, 35). This orderly progression and recovery are reminiscent of a Jacksonian epileptic march involving the motor cortex and suggests involvement of a body map as found in the primary motor cortex. Finally, recent views of the pathophysiology emphasize dystonia is a network disorder involving the basal ganglia, cerebellum, and motor cortices [for reviews see (1, 45, 46)].

Therefore, here we investigate this possibility using two-photon (2P) Ca^2+^ imaging and multiple single cell electrophysiological recordings before, during, and after an evoked episodic dystonia attack. Unexpectedly, the results suggest that motor cortical LFOs are not involved in the episodic dystonia. Instead, the findings show that the motor cortex coupling to limb movements decreases during the motor attack. Furthermore, the GSWD is nearly abolished during the episodic dystonia.

## Methods

### Animal care and husbandry

All animal studies were performed under protocols approved by the Institutional Animal Care and Use Committee of the University of Minnesota. Male and female *tg/tg* mice on a C57BL/6 background, as well as their wild type (WT) counterparts, were housed in University of Minnesota Research Animal Resources Facilities. Homozygous *tg/tg* mice were obtained by crossing a mouse line containing the *tg/tg* allele and closely linked semidominant allele Os, which causes oligosyndactalism. Genotyping of homozygous *tg/tg* mice was performed during weaning by confirming the absence of oligosyndactalism and was further verified *via* behavioral observation of episodic dystonia.

### Overview of experimental design

The study involved three separate experiments using three different cohorts of mice for each portion of the study: anesthetized, awake two-photon Ca^2+^ imaging, and awake electrophysiology recordings. Each procedure utilized a different cranial window preparation, as described below, and therefore could not be performed in the same set of animals. For the anesthetized experiments, we used 8 *tg/tg* (3 male, 5 female) and 5 WT (2 male, 3 female) mice. For the awake two-photon Ca^2+^ imaging, we recorded from 6 *tg/tg* (4 male, 2 female) and 4 WT (2 male, 2 female) mice, however one *tg/tg* mouse was excluded from the attack and recovery period due to unsuccessful limb tracking. Finally, for the awake electrophysiology recordings we used a total of 4 *tg/tg* (2 male, 2 female) and 4 WT (2 male, 2 female) mice.

### Anesthetized two-photon surgical preparation and image acquisition

Animals were anesthetized with acepromazine (2.0 mg/kg, i.p.) and urethane (2.0 mg/kg, i.p.), supplemented with 1.5 mg/kg urethane as needed. Once the animal was unresponsive to toe pinch, the skin covering the dorsal surface of the skull was removed, and a custom made imaging headplate was attached using a combination of Gel Superglue (Loctite) and Dental Cement (Dentsply Caulk). The animal then underwent a tracheotomy, was mechanically ventilated (120 breaths/min), and was then attached to a custom stereotax. Body temperature was maintained at 37°C using a feedback controlled heating pad. A 3 mm craniotomy was drilled over the left motor cortex (0 mm rostral, 1.5 mm lateral to bregma). Care was taken to avoid any bleeding and the dura was left intact. To image Ca^2+^ activity, 500 μM OGB-1 was then pressure injected using a fine-tipped glass micropipette at 3–4 sites in the craniotomy. An aqueous solution of 250 μM SR101 (Sigma) was placed on the surface of the brain to label astrocytes. Finally, the craniotomy was covered with a thin layer of agar and sealed with a glass coverslip and dental cement.

After surgical preparation was complete, the custom stereotax was secured to an xyz stage under a Leica SP5 multi-photon microscope with a MaiTai DeepSee laser tuned to 810 nm. Image series (3000 frames, 10 Hz) were taken over in randomly selected locations in M1. Some locations were imaged over multiple series to detect changes in LFOs over time. After imaging, mice were transcardially perfused using PBS and fixed with 4% paraformaldehyde.

### Chronic window implantation

Mice used for chronic imaging were implanted with a transparent skull window (47). Due to the notable phenotypic difference in head size between *tg/tg* and WT mice, custom windows for each strain were used [for fabrication methods, see (47)]. Each implant was designed such that motor, somatosensory, barrel, and the rostral portions of the visual cortices could be imaged. However, this study recorded only in the motor cortex. The implant consisted of two pieces: a 3D-printed polymethyl methacrylate (PMMA) frame with a polyethylene terephthalate (PET) film, and a titanium head plate to attach to a custom disk treadmill for head-fixed imaging.

Four hours prior to surgery start, mice were injected with 1 mg/kg slow-release Buprenorphene and 2 mg/kg Meloxicam. Adult mice, 3–8 months of age, were anesthetized using Isofluorane (5% induction, 0.75%–3% maintenance), and head-fixed in a stereotaxic frame. Body temperature was maintained at 37°C using a feedback controlled heating pad. The dorsal region of the skin was removed to expose the skull. Craniotomy coordinates were determined by tracing the implant on the exposed skull. Before the craniotomy was removed, three burr holes were drilled over the right motor cortex for delivery of AAV1-hSyn-GCaMP6f-WPRE (Penn Vector Core). At each injection site, 200 nL of virus was delivered (50 nL/min, 0.7 mm from the cortical surface) through a Nanofil syringe (World Precision Instruments) using an automated injector (UMP3, World Precision Instruments). The syringe was withdrawn after 5 min following each injection.

A single bone screw was placed over the cerebellum to give stability to the implant during attachment. Great care was taken to avoid blood loss during the skull removal. The PMMA portion of the implant was then attached with Vetbond (3M) and dental cement (C&B Metabond, Parkell Inc.). Once fully cured, the titanium head plate was attached to the implant using screws, and further secured with dental cement. A cap was affixed to the implant using Kwik-Sil (World Precision Instruments) to prevent damage to the brain from photobleaching as well as damage to the PET film from cage debris. Surgical attachment of the imaging window took roughly 4 h in total. Mice were allowed to recover from surgery for 3–4 days before experimentation.

### Awake two-photon image acquisition and data processing

Mice were head fixed to the disk treadmill and secured to the xyz stage under the SP5 microscope. The xyz positions were logged so that the precise location of each imaging field could be determined. A wavelength of 920 nm was used to excite GCaMP6f (48). Image series (6000 frames, 20 Hz) were acquired for 5 min. Behavioral data was collected using an IR-sensitive camera (DMK 33UP1300, Image Source) at 20 Hz. After recording 1 hour of baseline activity, episodic dystonia was evoked *via* injection of caffeine (15 mg/kg, I.P., in saline). Mice were imaged through the duration of the attack and 1 hour into recovery. The imaging software sent out an initial trigger pulse to an external pulse generator which then triggered the behavior camera acquisition at the same frame rate as the two-photon Ca^2+^ imaging. As the behavior cameras are IR sensitive, we are able to see the two-photon laser in the behavior images, and the behavior frames were then accurately aligned to the start and stop of the laser illumination.

The raw signal of individual neurons in M1 was obtained using ImageJ (NIH). In brief, xy motion artifact was corrected using the moco plugin (49), and cells were identified using the cell magic wand plugin (50). Raw fluorescence signals were extracted for each region of interest (ROI), imported into MATLAB 2016b (Mathworks) and detrended. Fluorescence data was converted into ΔF/F_0_, where F_0_ was defined as the 20th percentile of the overall fluorescence (51).

### Low-frequency oscillation analysis

To evaluate traces for evidence of LFOs, the spectral profile of each trace was obtained *via* the Welch’s power spectral density estimate in MATLAB (*pwelch*, 1000-point window, 100-point overlap, resolution of 4096). Setting the threshold for the high-power state was as described previously (41, 42). In brief, the threshold for high-power activity was set as 3 standard deviations above the WT mean in the 0.035–0.11 Hz band. In order to determine the degree of synchrony between high-power cells in a single time series, three analyses were performed. First, the local maxima of the frequency spectra of the cells were determined to probe maximally elevated frequency bands. If two local maxima were found within 10 data points of each other, the larger point was recorded. Second, the cross coherence between all high- and low-power traces in a time series was computed separately. The maximum coherence coefficient and frequency at which it occurred were extracted. To determine synchronicity in the time domain, cross correlation was computed separately for high- and low-power traces and the maximum correlation coefficient and time at which it occurred were extracted. Results from anesthetized data were used to set criteria within these parameters for LFO determination in the awake state.

### Simultaneous two-photon Ca^2+^ imaging and behavioral analysis during attack

To determine what changes in M1 paralleled attack behavior, we calculated the peak number, peak amplitude, coherence and correlation of traces in each stack using built in MATLAB functions. Behavior tracking was performed using DeepLabCut1.0 (52) with three points per paw. All movement analyses were performed using limb speed, for which position data was differentiated and converted to polar coordinates. Behavior coupling to Ca^2+^ data was determined by aligning ±1 s of behavior data to each Ca^2+^ peak. Periods of movement and rest were manually defined, and three standard deviations above the mean-aligned rest traces was used to define movement. If movement defined by these parameters occurred within 100 ms of the Ca^2+^ peak, cells were determined to encode movement.

### Awake electrophysiology surgical preparation and data acquisition

For experimentation, a separate cohort of *tg/tg* and WT C57BL/6 (Jackson Laboratories) were anaesthetized and prepared for surgery as described above. A 3–4 mm craniotomy was drilled over the motor cortex and a custom 3D-printed chamber was implanted over the craniotomy, affixed to the skull with dental screws and cement. The chamber was filled with sterile agarose gel and saline and sealed with Kwik-Sil. After surgery, mice recovered for 3–4 days prior to training. Mice were acclimatized to head-fixation on a custom freely moving recording wheel every other day for increasing durations to reduce the stress of being head-fixed and potential confounds with inducing the dystonic attack.

On recording days, up to 16 glass-gated platinum iridium microelectrodes were loaded into a multi-electrode array (System Eckhorn Microdrive, 100 μm spacing, Thomas Recording, GMBH), and were individually lowered. Electrophysiological signals were recorded at 24 KHz using a 32-channel pre-amp (PZ2–32, Tucker Davis Technologies) and RZ2 amplifier (Tucker Davis Technologies). Following 15–20 min of baseline activity, 15 mg/kg (i.p.) caffeine was injected to trigger the dystonic attack. Mice were continuously monitored for attack onset and duration (53).

### Electrophysiology data analysis

Raw signals were either filtered using a 150th order FIR low-pass filter (200 Hz) and down-sampled by a factor of 8 to obtain local field potentials (LFPs) or using a 150th order FIR bandpass filter (800–5,000 Hz) to obtain single units. Generalized spike-and-wave discharges (GSWDs) were isolated using a custom algorithm. Briefly, the short-time Fourier Transform (1 s window, 875 ms overlap, NFFT of 24,000) was computed and the product of the maximum powers for the first three resonant peaks (5–8, 13–17, and 19–24 Hz) were determined and thresholded against one standard deviation of the mode power. GSWDs were excluded if the duration was less than 1 s (54). Positive GSWD events were combined if two GSWDs occurred within 1 s of each other. Algorithm performance was approximately 95.9% accurate, with 0 false positives and 4.1% false negatives.

For single unit analysis, an amplitude threshold was selected to identify isolated action potentials. This was done using a standard outlier detection approach previously described (55), found to be 5 times the probable error of the overall signal. A waveform forming a 2-millisecond window around the identified peak was extracted. Three commonly used methods for feature extraction were applied to each identified waveform, include wavelet decomposition (56), Linear Discriminant Analysis (57, 58), and differential features including first and second derivatives (59). Features were first ordered by a modified Kolmogorov-Smirnov test for unimodality (56). For each feature, a KS-test was performed between the original distribution and a unimodal distribution of the same mean and standard deviation. Significant features (*p* < 0.05) were then ordered based on the obtained *p*-value. All significant features were then used to manually cluster the data.

Subsequently, unique units in each recording were subclassified as either interneurons or pyramidal cells using previously published methodology (60). Briefly, this was done by reducing the dimension of five parameters (mode interspike interval, mean firing rate, cell burst index, waveform asymmetry score, and waveform trough-to-peak latency) using t-distributed Stochastic Network Embedding (t-SNE) (61), a non-parametric method that computes the variability across each input dimension to assign a weight that retains the n-dimensional nearest-neighbor spatial arrangement of the data set. The output variables have arbitrary units but represent a weighted combination of the original features of the data. This classification system yielded two separable clusters with unique firing properties. To verify the classification, the firing properties of the cells were determined and compared to the literature values (62–64) including the firing rates and autocorrelation properties. Firing rhythmicity was characterized by the 2^nd^-order coefficient of variance (CV_2_), the ratio of the standard deviation to the mean squared, that shows the extent of variability in relation to the mean of the population (65, 66).

### Statistical evaluations

All statistics were performed in MATLAB and GraphPad Prism 8.0, and significance was set at *p* > 0.05. Data is reported as mean ± standard deviation. The mean correlation and coherence coefficients for *tg/tg* high-power, *tg/tg* low-power, and WT cells were compared using one-way ANOVA with Bonferroni *post hoc* test. Effect size (Cohen’s d) was calculated for all significant effects, and only significant effects with medium (>0.5, dashed lines) and large (>0.8, solid lines) effect sizes are shown in the figures. We report effect size to emphasize the magnitude of any changes, as small changes could result in significant but small effects given the large “n” values of the data sets. In the remainder of the manuscript, we only highlight comparisons that were both significant and passed these effect size criteria. All statistics are reported in Table 1.

## Results

### Low-frequency oscillations (LFOs) of movement kinematics during episodic dystonia in *tg/tg* mice

**FIGURE 1:**
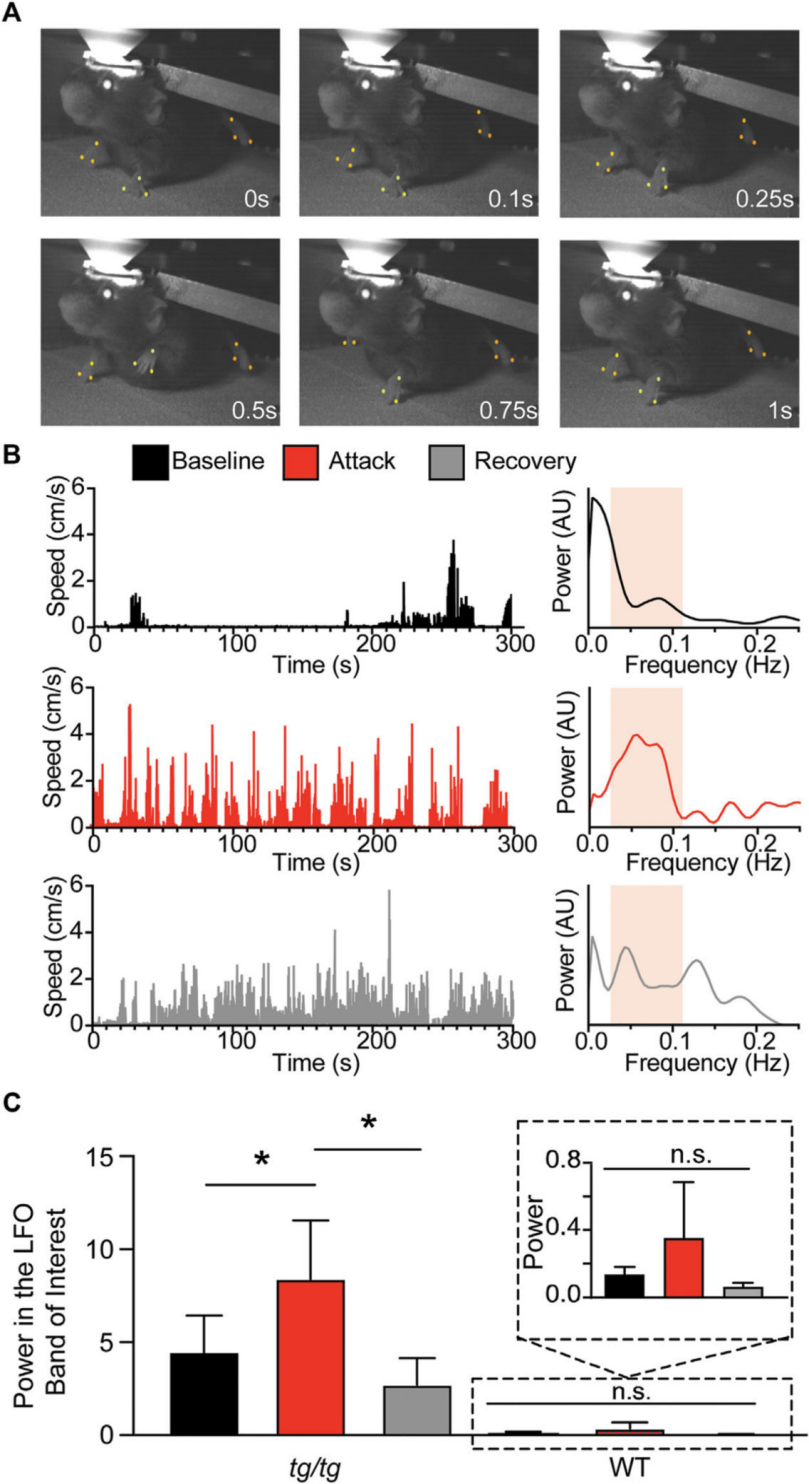
Presence of LFO in tg/tg limb kinematics during episodic dystonia. **(A)** Sample recording of a head-fixed *tg/tg* mousewith point tracking on right and left forelimbs and left hindlimb. **(B)** Speed analysis from the left hindlimb, calculated as the center of each paw from the three points indicated in ***A,*** from baseline, during, and recovery from episodic dystonia. Spectral analysis from each time period shows an increase in the LFO band of interest (0.035–0.11 Hz) during the attack. Band of interest is denoted by the beige shaded area in the power spectrum plots. **(C)** Quantification of the power from the left hindlimb showing significantly increased power differences between baseline-attack and attack-recovery. Inset shows zoomed in view of power fromWTmice. Data includes 6 episodic dystonia attacks from 5 *tg/tg* mice, and 4 sessions from 4 WTmice, all administered caffeine. Significant differences with large effect sizes indicated by a solid line.

To test our hypothesis that the LFOs observed in macroscopic brain imaging in the anesthetized *tg/tg* mouse are related to the attack, we performed high-speed video tracking of limb movements before, during, and after the attack (Figure 1A). Spectral analysis of DeepLabCut tracked limb movements showed increased rhythmicity of limb speed in the LFO band of interest (0.035–0.11 Hz) during the attack, as shown for example data (Figure 1B, red trace). This increase in power was significant and large in magnitude when compared to baseline and recovery across all mice, while wild-type mice showed no evidence of oscillation following caffeine challenge (Figure 1C). These results support earlier observations of LFO in the EMG activity of *tg/tg* during episodic dystonia (41) and suggest that the LFOs in the limb movement may be coupled to LFOs in the cerebral cortex and/or cerebellar cortex (42).

### Ca^2+^ imaging of motor cortical neurons reveals high-power LFOs in the anesthetized *tg/tg* mice but not during awake behavior or attack

**FIGURE 2:**
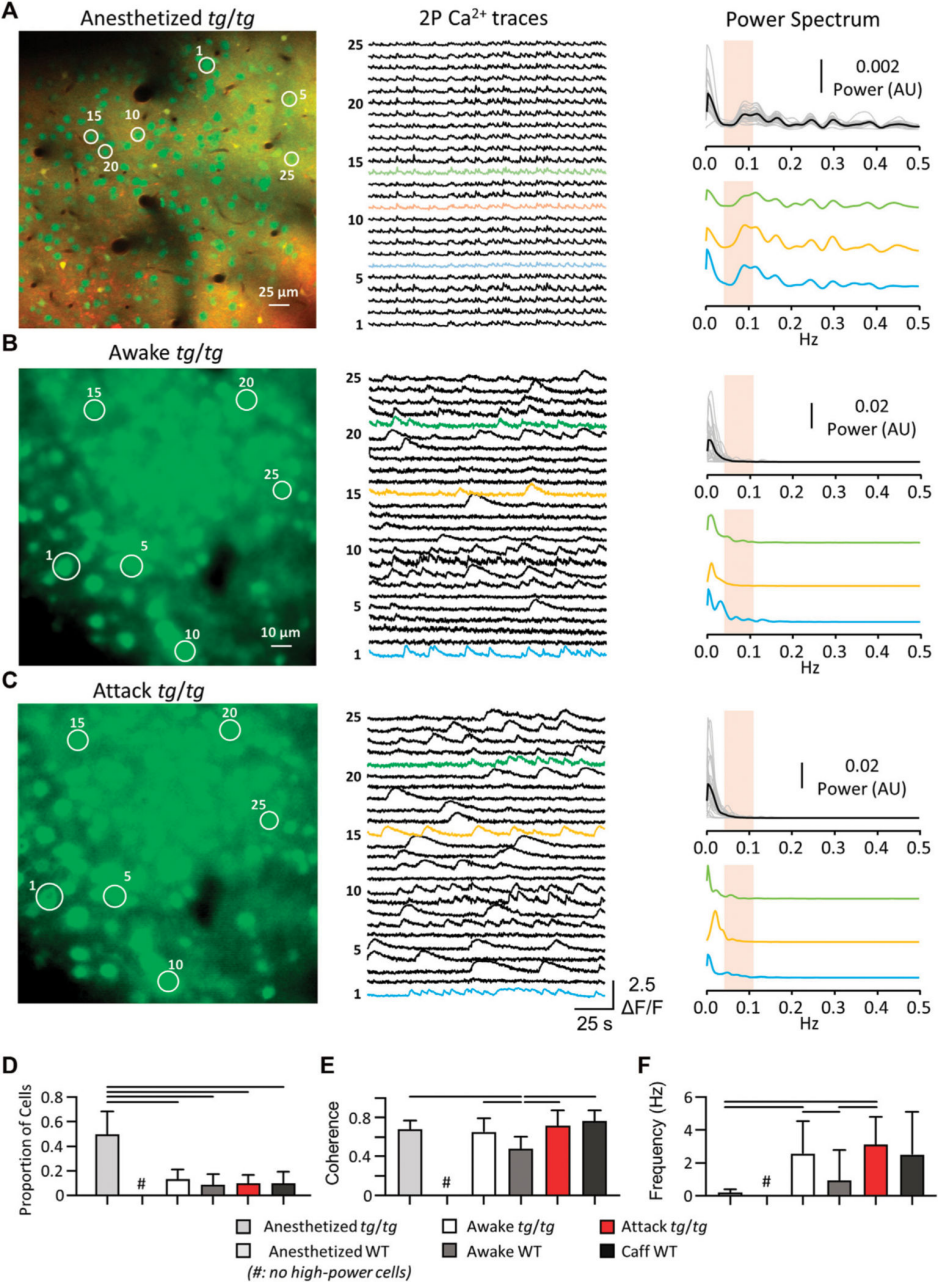
Single-cell Ca^2+^ activity reveals LFO present in *tg/tg* mice only under anesthesia. **(A)** Sample OGB-1/SR101 (green/red, respectively; overlap in yellow) recording from a *tg/tg* mouse under urethane anesthesia, with individual single-cell Ca^2+^ activity and spectral density plots (each cell power spectra in grey, mean in black) for the color-coded traces. **(B)** Sample GCaMP6f recordings and spectral plots from *tg/tg* motor cortex during awake, baseline behavior. **(C)** Sample GCaMP6f recording and spectral plots from the same mouse and cells as in ***B*** during an evoked episodic dystonia attack. Band of interest is denoted by the beige shaded area in the power spectrum plots in **(A–C). (D)** Proportion of cells that experience the high-power state. **(E)** Changes in the largest magnitude-squared coherence between cells in different animals and states. **(F)** Quantification of the frequency at maximum coherence. For **(D,F)**, significant differences with large (solid line) effect sizes are shown. Data collected from 8 *tg/tg* (3 male, 5 female) and 5 WT (2 male, 3 female) mice for the anesthetized recordings, and 6 *tg/tg* (4 male, 2 female) and 4 WT (2 male, 2 female) mice for the awake recordings.

Previous studies of the LFOs in *tg/tg* mice were performed under anesthesia using flavoprotein imaging (41, 42), which uses metabolic activity as a surrogate of cellular activation. However, flavoprotein imaging cannot distinguish signals from different cell types or the activity of single neurons. To test whether LFOs occur at the neuronal level in the *tg/tg* mouse, we first performed single cell two-photon (2P) imaging under two conditions in layer II/III of the primary motor cortex (M1): 1) under urethane-anesthesia using the Ca^2+^ indicator OGB-1 (Figure 2A); and 2) in awake mice using virally injected GCaMP6f (Figures 2B, C; see Methods). Example single cell activity and spectral analysis are shown for a sample of cells identified in the image (Figure 2A, right). Using the previously established criteria of high-power LFO activity in anesthetized *tg/tg* mice (3 standard deviations above the mean of WT mice (Cramer et al., 2015)), we identified 49.36 ± 19.01% of cells in each of 8 mice recorded with high-power activity, while no cells recorded in the anesthetized WT mouse that exhibited high-power LFO activity (Figure 2D). Thus, the anesthetized WT condition was excluded from the remainder of LFO analysis as there were no cells with high-power LFOs identified. Analysis of the activity of the *tg/tg* high-power cells in the frequency domain reveals a high degree of maximum coherence (Figure 2E). Furthermore, the mean peak frequency of the *tg/tg* high-power cells (0.23 ± 0.14 Hz) is within the LFO band of interest.

We next performed awake two-photon imaging of head-fixed *tg/tg* mice before, during, and following caffeine (15 mg/kg I.P.) induced episodic dystonia. Example single cell activity and spectral analysis are shown for a sample of cells identified in the image (Figures 2B, C, right). Using the same LFO detection criteria as in the anesthetized recordings, we identified less than 12% of cells with high-power activity in awake and attack *tg/tg* conditions (Figure 2D). While the coherence amplitude was high across high-power cells from the different groups, with some differences (Figure 2E), the maximum coherence was in the LFO band of interest only for neurons recorded in the anesthetized *tg/tg* mouse (0.23 ± 0.14 Hz, Figure 2F). Taken together, these results confirm that LFOs are present in *tg/tg* M1 neurons and the Ca^2+^ transients in these cells are highly synchronized under anesthesia. Injections of caffeine in WT mice did not result in an increase in LFOs in motor cortex neurons, therefore caffeine itself does not produce the oscillations. However, in awake, behaving *tg/tg* mice, we observed only a small percentage of neurons with LFOs, in either the baseline or episodic dystonia state.

### Neuronal firing rate and variability do not change during episodic dystonia

**FIGURE 3:**
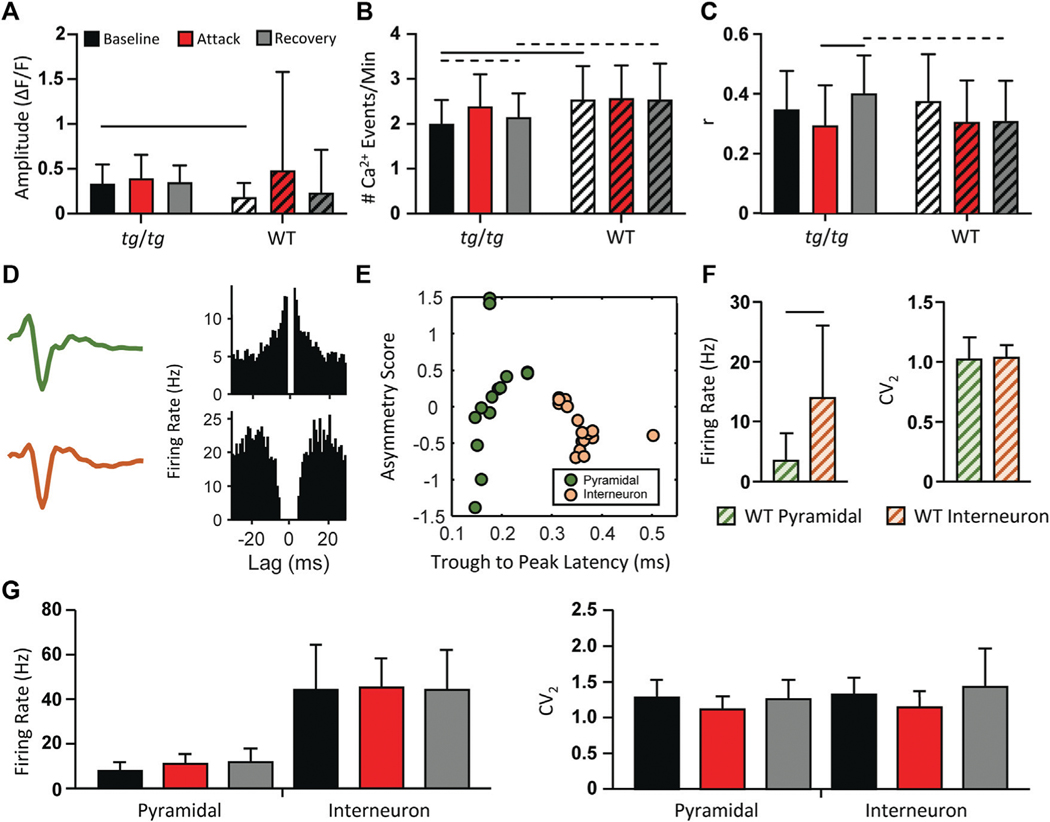
Minimal changes in *tg/tg* motor cortex single-cell Ca^2+^ and extracellular single-unit activity between baseline, attack, and recovery. **(A)** Quantification of ΔF/F GCaMP6f amplitude from caffeine injected *tg/tg* and WT mice. **(B)** Number of Ca^2+^ events per minute. **(C)** Average between-cell correlation (r) of ΔF/F activity. **(D)** Sample extracellular single-unit waveform and autocorrelogram of their firing rate from a putative pyramidal (green, top) and inhibitory (orange, bottom) cell in a WT mouse. **(E)** Visualization of metrics used for WT pyramidal and interneuron classification. **(F)** Quantification of firing rate and CV_2_ differences between WT pyramidal and interneurons. **(G)** Firing rate (left) and CV_2_ (right) quantification in *tg/tg* mice reveal no significant change in either parameter from pyramidal or interneurons between baseline, attack, or recovery. For **(A–C, F),** significant differences with medium (dashed line) or large (solid line) effect sizes are shown. Data collected from 6 *tg/tg* (4 male, 2 female) and 4 WT (2 male, 2 female) mice for the awake Ca^2+^ recordings, and from 4 *tg/tg* (2 male, 2 female) and 4 WT (2 male, 2 female)mice for the awake electrophysiology recordings.

Although the activity of *tg/tg* M1 neurons did not display synchronized LFOs during episodic dystonia, we examined if there were any other changes in Ca^2+^ activity. No significant changes with a large enough effect size were observed in the amplitude of Ca^2+^ events in *tg/tg* mice throughout the recordings (Figure 3A). There was a significant increase (large effect size) in both the amplitude (Figure 3A) and number (Figure 3B) of Ca^2+^ events during baseline between *tg/tg* and WT mice, likely attributed to the mutated P/Q-type Ca^2+^ channels. The number of Ca^2+^ events did remain significantly larger (medium effect size) in the recovery period compared to baseline in *tg/tg* mice (Figure 3B), and an increase in the correlation of cells in local M1 networks following but not during the attack (Figure 3C), suggesting some long-lasting alterations in M1 cells following an episodic dystonia attack. Taken together, these results suggest that these modest, non-specific changes in Ca^2+^ event metrics in layers II/III M1 neurons may be insufficient to attribute a major role for the primary motor cortex in the episodic dystonia.

As 2P Ca^2+^ imaging is predominantly restricted to the upper layers of the cerebral cortex, does not directly measure neural spiking, and is not cell type specific unless using targeted expression systems, we performed multielectrode extracellular recordings in the motor cortex before, during, and following the episodic dystonia in *tg/tg* mice. To first examine for neuronal specific type specific changes, pyramidal cells and inhibitory interneurons were classified in WT mice using the established approaches detailed in the Methods (Figures 3D–F). Applying these classification criteria to the neurons recorded in *tg/tg* mice revealed 38 excitatory and 84 inhibitory neurons during baseline, 33 and 68 during the attack, and 36 and 52 during recovery. Due to the nature of the large dystonic movements, some cells were lost during the attack and into the recovery period. Importantly, neither the firing rate or CV_2_ of either cell type in *tg/tg* mice significantly changed during the episodic dystonia. The lack of any electrophysiological changes not only confirms, but extends the 2P imaging results, and suggest a lack of motor cortical involvement in the dystonic attack.

### Spike triggered coupling to kinematics decreases during episodic dystonia

**FIGURE 4:**
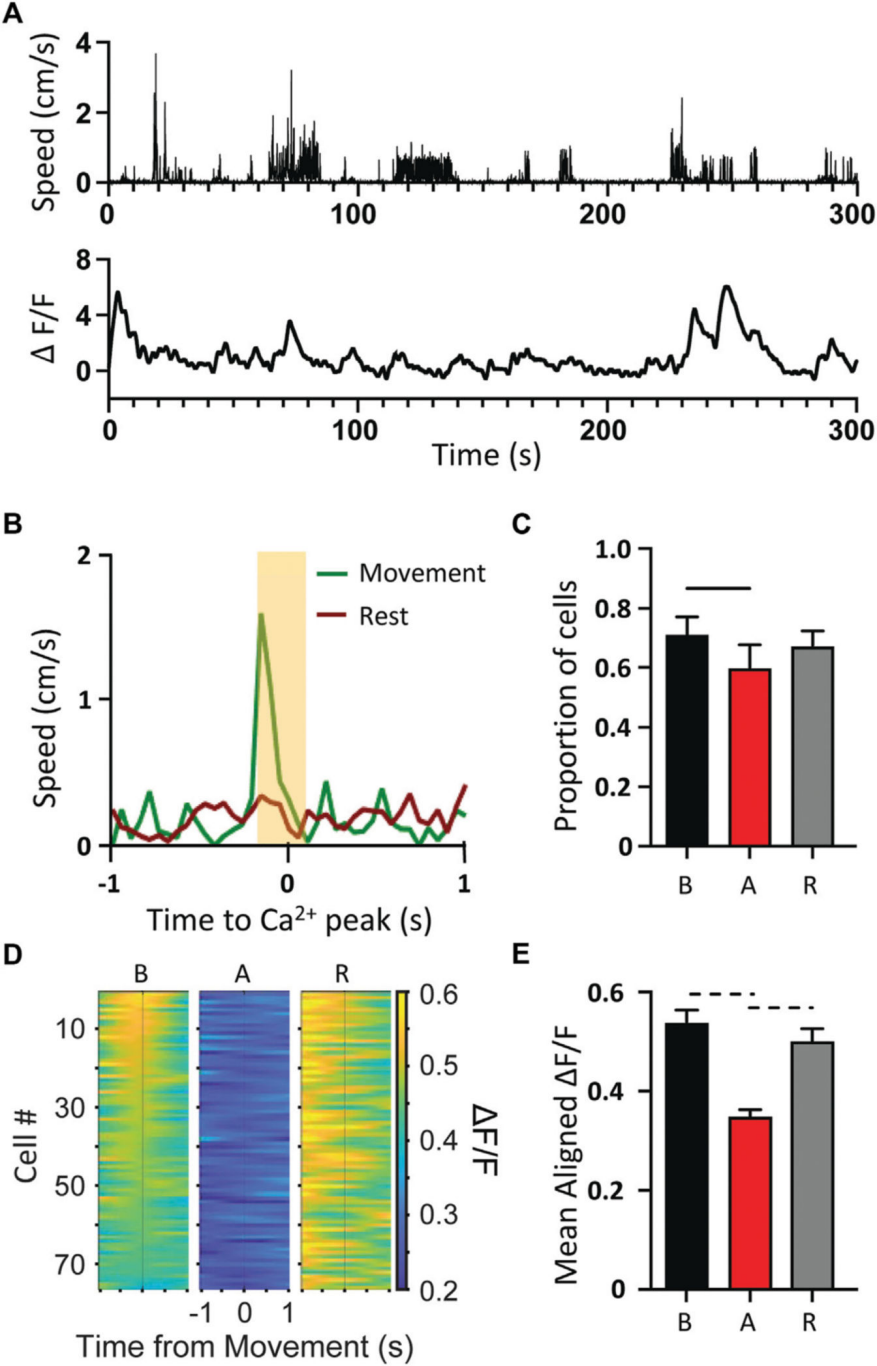
Decoupling of Ca^2+^ activity to movement kinematics during *tg/tg* attack. **(A)** Sample movement speed of forelimb (top) and 2P single-cell Ca^2+^ ΔF/F trace (bottom) from a *tg/tg* mouse during baseline conditions. **(B)** Example trace from classified “movement” cell. **(C)** Quantification of the proportion of movement cells from baseline to attack and recovery. **(D)** Movement onset aligned PSTH for left forelimb for representative session. **(E)** Quantification of average fluorescence 200 ms surrounding movement onset for all attacks. For **(C, E),** significant differences with medium (dashed line) or large (solid line) effect sizes are shown. Data collected from 5 *tg/tg* (3 male, 2 female) mice.

The lack of overt, dystonic-like changes in either the Ca^2+^ activity or the spike firing statistics of M1 neurons of the *tg/tg* mouse suggests that M1 may partially decouple from the downstream movements during the dystonic attack. To test this hypothesis, we performed a spike triggered behavior inspired approach in which for each neuron we aligned forelimb kinematics (Figure 4A, top) to the Ca^2+^ peaks (Figure 4A, bottom) during periods of movement and rest (Figure 4B). Cells that had limb speed during movement that was 3 standard deviations above the rest mean 100 ms on either side of Ca^2+^ peaks were considered ‘movement cells’ and assumed to encode limb behavior. This is illustrated for an example movement cell, showing that the Ca^2+^ peak was coupled to and led the limb speed (Figure 4B). The proportion of movement cells significantly decreased during the dystonic attack and significantly increased during the recovery period, but not quite returning to baseline levels (Figure 4C). Aligning the Ca^2+^ ΔF/F to movement onset revealed a strong relationship with Ca^2+^ activity to movement during the baseline period, that significantly decreased during the attack (Figures 4D,E) and recovered to baseline levels in the recovery period, suggesting that M1 partially decouples from behavioral output during the motor attack.

### Generalized spike-and-wave discharges are abolished during episodic dystonia

**FIGURE 5:**
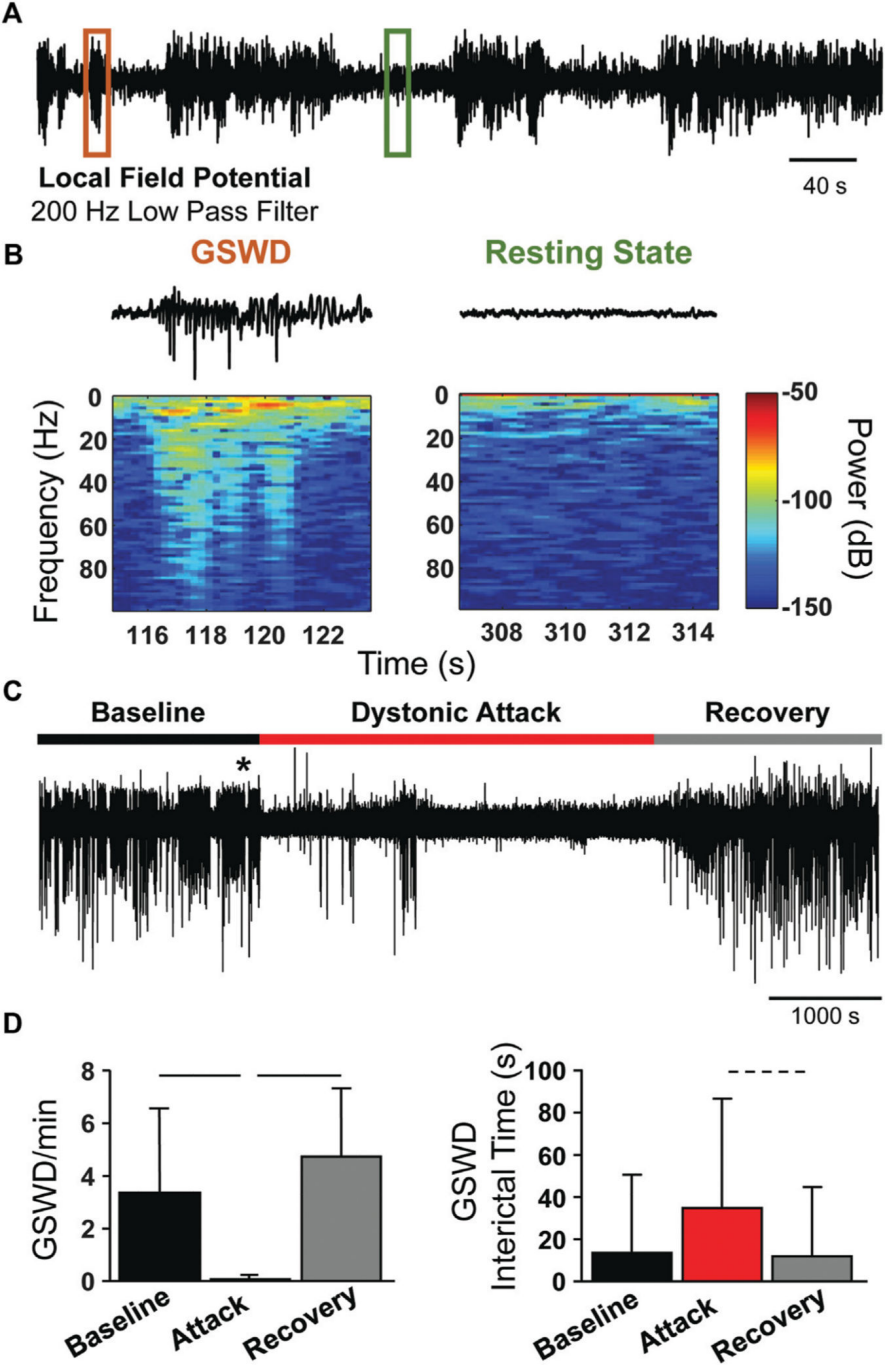
Loss of GSWDs during episodic dystonia attack in *tg/tg* mice. **(A)** Example motor cortex LFP trace from an awake *tg/tg* mouse. **(B)** Zoomed in views of the LFP trace from the color-coded regions in **(A),** showing an example GSWD and resting period (top) and the corresponding spectral content (bottom). **(C)** Continuous motor cortex LFP recording from a *tg/tg* mouse during baseline, caffeine-evoked episodic dystonia attack, and recovery. * denotes time of caffeine injection. **(D)** Quantification of GSWD occurrence rate (left) and interictal time (right) during all recordings. Significant differences with medium (dashed line) or large (solid line) effect sizes are shown. Data collected from 4 *tg/tg* (2 male, 2 female) and 4 WT (2 male, 2 female) mice.

The *tg/tg* mice exhibit generalized spike-and-wave discharges (GSWDs) that are an underlying mechanism of absence seizures and one of the major phenotypes of the mutation in the *cacna1a* gene. However, the relationship between GSWDs and the episodic dystonia are not well understood. As the Ca^2+^ imaging shows that M1 is partially decoupled from behavior, we reasoned that the GSWDs in the cerebral cortex may be similarly affected. Therefore, we recorded the local field potentials (LFPs) from *tg/tg* mice and determined the presence and timing of GSWDs (Figure 5A) in relation to a dystonic attack. A GSWD event is characterized by the high amplitude deflections in the LFP trace and increased power (Figure 5B) compared to the resting state (no GSWDs present). During the dystonic attack, GSWDs were almost completely abolished. As shown for a representative LFP recording during the course of a caffeine-evoked episodic dystonia attack, there are negligible GSWDs during the attack (Figure 5C), while prominent GSWDs occur during both the baseline and recovery periods.

Quantification of the average GSWD rate (per minute) during the baseline, attack, and recovery periods show a marked decrease in GSWD rate during the attack, compared to baseline and recovery periods (Figure 5D, left). Correspondingly, the GSWD interictal time significantly increased during the attack period (Figure 5D, right), but did not quite reach a medium effect size (Cohen’s d = 0.47), likely due to the extremely small number of GSWD events during the attack. The loss of GSWDs, as well as the uncoupling of Ca^2+^ activity to movement kinematics, suggests that the episodic dystonic attack induces a different brain state in *tg/tg* mice.

## Discussion

Our past work highlighting the presence of transient LFOs in the cerebral cortex of the anesthetized *tg/tg* mouse that are markedly reduced by ACTZ and 4-AP, two of the major treatments for EA2 (Cramer et al., 2015), lead us to hypothesize a central role for the these rather unique oscillations in the dystonic attack in this EA2 mouse model. Here we tested this hypothesis, using 2P Ca^2+^ imaging and electrophysiological recordings of single neurons, as well as LFPs, before, during, and following caffeine-triggered dystonic attacks.

### Presence of LFOs in limb kinematics during dystonic attack

Further extending our hypothesis that brain LFOs are central to the dystonic attack in awake, behaving *tg/tg* mice, we observed that limbs movements are characterized by frequencies within the cortical LFO band of interest throughout the dystonic attack. This finding confirms earlier observations of EMG activity at these low frequencies (41). As a similar magnitude increase did not occur in WT mice, even when caffeine was administered, this change is likely attributable to the caffeine-triggered neural mechanisms that underlie the *tg/tg* attack, but not the caffeine itself. Given that neuronal firing in M1 has long been linked to limb kinematics [for reviews see (67, 68)], we hypothesized that the LFOs would be reflected in M1 activity during the motor attack in awake, behaving *tg/tg* mice.

### Two-photon Ca^2+^ imaging during an episodic dystonic attack

Previous wide-field imaging studies using flavoprotein as a surrogate for neuronal activation observed the LFOs in cerebellum and cerebral cortex (41, 42). These studies were unable to attribute this activity to single cells or specific subtypes of cells/neurons. Here, we observed LFOs in the Ca^2+^ transients of individual layer II/III M1 neurons in the anesthetized *tg/tg* mouse. This cellular level activity exhibited high spectral coherence in the LFO band of interest, consistent with earlier, wide-field flavoprotein optical recordings of LFOs in the cerebral cortex (42). This confirms that LFOs are present at the single cell level in anesthetized *tg/tg* mice.

Surprisingly, we did not find evidence for LFOs in the Ca^2+^ fluorescence signals in M1 neurons in the awake *tg/tg* mouse, at rest or during a dystonic attack. In addition to assessing for the presence of LFOs, we evaluated changes in the number, amplitude, and correlation of Ca^2+^ transients in relation to a dystonic attack. No significant changes or effect sizes were observed in either the amplitude or number of Ca^2+^ events comparing baseline to the attack state, with only a medium effect size in the number of Ca^2+^ events between baseline and recovery, and a large effect size in the between-cell correlation from the attack to recovery. In our view, these changes in M1 neurons are not consistent with the magnitude of the abnormal limb movements in the LFO band of interest during the attack. Taken together, the 2P imaging results do not support our original hypothesis that LFOs in the activity of M1 neurons have a major role in the episodic dystonia in the *tg/tg* mouse.

### LFP and single cell recordings during a dystonic attack

While we did not observe LFOs or large changes in the Ca^2+^ events in M1 neurons during the episodic dystonia, our 2P imaging was restricted to recording neurons in layers II/III. There is a possibility that layer V output neurons would exhibit large changes in firing that contribute to the motor attack. Therefore, we recorded ensembles of M1 neurons that spanned all cortical layers, sorted into excitatory and inhibitory classes based on discharge properties, and tested for changes in firing statistics. Notably, both classes of neurons showed no significant changes in firing statistics during the dystonic attack, further underscoring our interpretation of the Ca^2+^ data and highlighting a remarkable lack of activity changes in M1 neurons during the dystonic attack in *tg/tg* mice.

### M1 activity decouples from limb kinematics during dystonic attack

Given a lack of pronounced firing and activity changes across M1 layers in *tg/tg* mice during the dystonic attack, despite very pronounced changes in limb kinematic patterns, we performed additional analysis to quantify the relationship of M1 activity to limb motor output before, during, and after the dystonic attack. Aligning either movement onset to identified Ca^2+^ peaks, or aligning Ca^2+^ activity to identified movement onset revealed a significant decrease in the number of movement cells and Ca^2+^ activity surrounding movement onset during the attack, respectively. Taken together, these findings suggest that M1 activity decouples from movement output during the dystonic attack. This is consistent with EA2 patient reports that although they are conscious, the dystonic limb movements are involuntary (69). While the findings support a decoupling of the motor cortex from the motor output, we acknowledge that other explanations need to be considered. One possibility is that non-cortical structures are driving the dystonia, providing a powerful drive to the spinal cord and alpha motor neuron pools that overwhelms any descending inputs from M1. Another explanation is that afferent input to M1 is disrupted, not the output. Additional studies are needed to test the various possibilities.

### Loss of GSWD during dystonic attack

We also investigated a second hallmark of the *tg/tg* phenotype, GSWD. During the attack period, we consistently observed a near complete loss of the GSWDs that returns to baseline-like levels during the recovery period. It is well established that the thalamo-cortical circuitry plays a critical role in both GSWD and absence seizures [for reviews see (70, 71)]. In the *tg/tg* mouse, cerebellar involvement with GSWDs has been shown by phase-locking of nuclear neuron firing and GSWDs in the cerebral cortex. Further, GSWD can be modulated by the cerebellum, as optogenetic inhibition of the cerebellar nuclei to the thalamus suppresses GSWDs (54, 72). When coupled with the present observations, GSWD loss during the episodic dystonia suggests that connectivity between the cerebellum and motor cortex, *via* the thalamus, is causally disrupted during the attack.

### Distinct brain and behavioral states in the *tg/tg* mouse

The present results highlight disparate brain and behavior states within the *tg/tg* mouse model of EA2. Mice display a baseline behavioral state marked by GSWDs and absence seizures, mild ataxia, and coupling between M1 activity and limb behavior. Under anesthesia, LFOs are present throughout the dorsal cerebral cortex and the cerebellar cortex (41, 42). The attack state is marked by notable differences in behavior and brain activity. Behaviorally, animals are racked with strong dystonic limb movements that are characterized by LFOs. During a dystonic attack, the cerebellar cortex exhibits LFOs, while neuronal activity in M1 changes very little. Furthermore, the normally ubiquitous GSWDs are largely absent and M1 neuronal activity is significantly decoupled from motor output. Following the motor attack, mice return to the baseline behavioral state. Therefore, there are multiple brain states due to the mutation in the *cacna1a* gene in the mouse that underlie the behavioral phenotypes.

One of these brain states is the dramatic high-power LFOs in cerebral cortex of anesthetized *tg/tg* mice, but these cortical LFOs are not present in the awake animal or during the dystonic attack. These oscillations are consistent with observations that anesthesia induces highly correlated, brain-wide, low-frequency oscillations in the mouse (73, 74). As shown recently in wide-field Ca^2+^ imaging, these oscillations include waves of activity that spread across the dorsal cerebral cortex (74). In wild-type mice, the frequency of these oscillations ranges from 0.75 to 2 Hz, compared to the much lower frequencies observed in *tg/tg* mice. We speculate that the mutation in the P/Q-type Ca^2+^ channel shifts the excitability of neurons in the cerebral cortex to synchronize at these very low frequencies, the same frequencies observed in the limb movements.

The second brain state is the loss of both GSWDs and decoupling of M1 from the limb movements during the dystonic attack. However, during the attack, LFOs are present in the cerebellar cortex and increase in coherence with the limb EMG activity (41). Therefore, the cerebral cortex and cerebellum have fundamentally different excitatory states, and therefore roles, during the episodic dystonia. In the cerebellar cortex, the synchronization and propagation of the LFOs occur over 30–120 min (41), similar to the duration of the episodic dystonia (29, 31, 53). We have hypothesized that the oscillations in Purkinje cell output will be transmitted downstream to the cerebellar nuclei and their targets in the brainstem that project to the spinal cord (Campbell and Hess, 1998), potentially supplying a powerful input to muscles that is expressed as the episodic dystonia. As described above, the loss of GSWDs upstream in the cerebral cortex may also be due to the abnormal cerebellar output. A major remaining question is whether the lack of LFOs in the cerebral cortex and decoupling from the dystonic movements actually facilitates the abnormal movements by allowing the cerebellar output to modulate downstream pathways without interference from descending cerebral cortical activity.

Support has increased for cerebellar involvement in several dystonias [for reviews see (45, 46)] and would be the focus of future investigations. We would determine if the LFOs in the cerebellar cortex are driving the episodic dystonia, by testing whether blocking cerebellar cortical LFOs stopped an ongoing motor attack. A complementary test would be to determine if initiating LFOs in the cerebellar cortex generates an attack. It would be important to test if the two main treatments for EA2, 4-AP and ACTZ, when applied to the cerebellum block the LFOs and control the attacks. Further investigations would assess how the different classes of neurons in the cerebellum are modulated during an attack, as well as the involvement of mossy fiber inputs and climbing fiber input from the inferior olive. Also, probing cerebellar output pathways during the episodic dystonia, including the cerebellar nuclei and their brainstem targets, would be important to understand how the LFOs are propagated. If cerebellar LFOs could be firmly established as the origin of the motor attacks, this would suggest future therapeutic targets.

Regardless of the mechanism that provided the switch between baseline and attack states, this work establishes a new role for M1 in the generation of the dystonic attack in *tg/tg* mice. Future interventions to correct the *tg/tg* dystonic phenotype (and perhaps EA2 itself) may hinge upon preventing the emergence of the ‘attack’ brain phenotype that includes decoupling of M1 from the motor output.

## Figures and Tables

**TABLE 1:** Statistical testing results and effect sizes.

	Tukey’s multiple comparisons test	Adjusted *p*-value	Effect size (Cohen’s d)
Figure 1C	Baseline *tg/tg* vs. Attack *tg/tg*	0.03	0.57
	Baseline *tg/tg* vs. Recovery *tg/tg*	0.92	0.39
	Attack *tg/tg* vs. Recovery *tg/tg*	0.02	0.89
	Baseline WT vs. Caff WT	0.72	Ns
	Baseline WT vs. Recovery WT	0.96	Ns
	Caff WT vs. Recovery WT	0.56	Ns
	*n* = 6 attacks from 5 *tg/tg* mice, 4 caffeine administrations in 4 WT mice		
Figure 2D	Anesthetized *tg/tg* vs. Awake *tg/tg*	<0.0001	2.5
	Anesthetized *tg/tg* vs. Awake WT	<0.0001	2.81
	Anesthetized *tg/tg* vs. Attack *tg/tg*	<0.0001	2.68
	Anesthetized *tg/tg* vs. WT caff	<0.0001	2.65
	Awake *tg/tg* vs. Awake WT	0.78	Ns
	Awake *tg/tg* vs. Attack *tg/tg*	0.97	Ns
	Awake *tg/tg* vs. Caff WT	0.98	Ns
	Awake WT vs. Attack *tg/tg*	>0.99	Ns
	Awake WT vs. Caff WT	>0.99	Ns
	Attack *tg/tg* vs. Caff WT	>0.99	Ns
Figure 2E	Anesthetized *tg/tg* vs. Awake *tg/tg*	0.81	Ns
	Anesthetized *tg/tg* vs. Awake WT	<0.0001	1.93
	Anesthetized *tg/tg* vs. Attack *tg/tg*	0.65	Ns
	Anesthetized *tg/tg* vs. WT caff	0.2	Ns
	Awake *tg/tg* vs. Awake WT	<0.0001	1.4
	Awake *tg/tg* vs. Attack *tg/tg*	0.083	Ns
	Awake *tg/tg* vs. Caff WT	0.028	Ns
	Awake WT vs. Attack *tg/tg*	<0.0001	1.76
	Awake WT vs. Caff WT	<0.0001	2.47
	Attack *tg/tg* vs. Caff WT	0.83	Ns
Figure 2F	Anesthetized *tg/tg* vs. Awake *tg/tg*	0.044	1.70
	Anesthetized *tg/tg* vs. Awake WT	0.96	Ns
	Anesthetized *tg/tg* vs. Attack *tg/tg*	0.0078	2.45
	Anesthetized *tg/tg* vs. WT caff	0.15	Ns
	Awake *tg/tg* vs. Awake WT	0.0009	0.87
	Awake *tg/tg* vs. Attack *tg/tg*	0.71	Ns
	Awake *tg/tg* vs. Caff WT	>0.9999	Ns
	Awake WT vs. Attack *tg/tg*	<0.0001	1.25
	Awake WT vs. Caff WT	0.13	Ns
	Attack *tg/tg* vs. Caff WT	0.93	Ns
	*Anesthetized WT had 0 high power cells		
	Number of recordings with high power cells/number of high-power cells		
	Anesthetized *tg/tg*: 8 recordings/81 cells		
	Anesthetized WT: 0 recordings/0cells (13 overall recordings)		
	Awake *tg/tg*: 22 recordings/574 cells		
	Awake WT: 11 recordings/222 cells		
	Attack *tg/tg*: 6 recordings/233 cells		
	WT caffeine: 4 recordings/57 cells		
Figure 3A	Baseline *tg/tg* vs. Attack *tg/tg*	0.14	Ns
	Baseline *tg/tg* vs. Recovery *tg/tg*	>0.9999	Ns
	Attack *tg/tg* vs. Recovery *tg/tg*	0.59	Ns
	Baseline WT vs. Caff WT	<0.0001	0.38
	Baseline WT vs. Recovery WT	0.91	Ns
	Caff WT vs. Recovery WT	<0.0001	0.3
	Baseline *tg/tg* vs. Baseline WT	<0.0001	0.8
	Attack *tg/tg* vs. Caff WT	0.026	0.11
	Recovery *tg/tg* vs. Recovery WT	0.001	0.32
Figure 3B	Baseline *tg/tg* vs. Attack *tg/tg*	<0.0001	0.16
	Baseline *tg/tg* vs. Recovery *tg/tg*	0.0004	0.74
	Attack *tg/tg* vs. Recovery *tg/tg*	<0.0001	0.41
	Baseline WT vs. Caff WT	>0.9999	Ns
	Baseline WT vs. Recovery WT	>0.9999	Ns
	Caff WT vs. Recovery WT	>0.9999	Ns
	Baseline *tg/tg* vs. Baseline WT	<0.0001	1.03
	Attack *tg/tg* vs. Caff WT	0.0002	0.21
	Recovery *tg/tg* vs. Recovery WT	<0.0001	0.76
Figure 3C	Baseline *tg/tg* vs. Attack *tg/tg*	<0.0001	0.41
	Baseline *tg/tg* vs. Recovery *tg/tg*	<0.0001	0.42
	Attack *tg/tg* vs. Recovery *tg/tg*	<0.0001	0.82
	Baseline WT vs. Caff WT	<0.0001	0.48
	Baseline WT vs. Recovery WT	<0.0001	0.46
	Caff WT vs. Recovery WT	>0.9999	Ns
	Baseline *tg/tg* vs. Baseline WT	0.023	0.2
	Attack *tg/tg* vs. Caff WT	0.97	Ns
	Recovery *tg/tg* vs. Recovery WT	<0.0001	0.71
	For Figures 3A-C: *n* = 692 *tg/tg* cells from 6 animals, 351 WT cells from 4 animals		
Figure 3F	WT Firing Rate Pyr vs. Int	0.0036	1.14
	WT CV2 Pyr vs. Int	0.4463	Ns
	*n*= 16 pyramidal cells, 19 interneurons		
Figure 3G	Firing Rate Int Baseline vs. Attack	0.56	Ns
	Firing Rate Int Baseline vs. Recovery	0.93	Ns
	Firing Rate Int Recovery vs. Attack	0.64	Ns
	Firing Rate Py Baseline vs. Attack	0.57	Ns
	Firing Rate Py Baseline vs. Recovery	0.63	Ns
	Firing Rate Py Recovery vs. Attack	0.46	Ns
	CV2 Int Baseline vs. Attack	0.37	Ns
	CV2 Int Baseline vs. Recovery	0.75	Ns
	CV2 Int Recovery vs. Attack	0.46	Ns
	CV2 Py Baseline vs. Attack	0.48	Ns
	CV2 Py Baseline vs. Recovery	>0.99	Ns
	CV2 Py Recovery vs. Attack	0.35	Ns
Figure 4C	Baseline vs. Attack	0.01	1.64
	Baseline vs. Recovery	0.51	Ns
	Attack vs. Recovery	0.069	Ns
Figure 4E	Baseline vs. Attack	<0.0001	0.54
	Baseline vs. Recovery	0.4	Ns
	Attack vs. Recovery	<0.0001	0.51
	*n* = 334 cells from 5 animals		
Figure 5D	GSWD/min Baseline vs. Attack	<0.001	1.27
	GSWD/min Baseline vs. Recovery	0.38	Ns
	GSWD/min Recovery vs. Attack	<0.001	2.38
	Interictal Time Baseline vs. Attack	<0.001	0.47
	Interictal Time Baseline vs. Recovery	0.8	Ns
	Interictal Time Recovery vs. Attack	<0.001	0.53

## Data Availability

The raw data supporting the conclusion of this article will be made available by the authors, without undue reservation.
